# Characteristics and function of a novel cystatin gene in the pine wood nematode *Bursaphelenchus xylophilus*

**DOI:** 10.1242/bio.042655

**Published:** 2019-09-11

**Authors:** Qi Xue, Xiao-Qin Wu

**Affiliations:** 1 Co-Innovation Center for Sustainable Forestry in Southern China, College of Forestry, Nanjing Forestry University, Nanjing, Jiangsu 210037, China; 2 Jiangsu Key Laboratory for Prevention and Management of Invasive Species, College of Forestry, Nanjing Forestry University, Nanjing, Jiangsu 210037, China

**Keywords:** Cystatin, Expression, Development, Pathogenicity

## Abstract

*Bursaphelenchus xylophilus* is the pathogen that causes pine wilt disease (PWD). The disease has caused significant economic losses and damage to forests. However, the pathogenic mechanism of *B. xylophilus* remains unclear. Cystatins are involved in various biological processes where they regulate normal proteolysis and also play a role in pathogenicity, but their functions in *B. xylophilus* are unknown. Therefore, we cloned the full-length cDNA of a cystatin gene of *B. xylophilus* (*Bx-cpi-1*) by rapid-amplification of cDNA ends and analyzed its characteristics with bioinformatic methods. *In situ* mRNA hybridization analyses showed that transcripts of *Bx-cpi-1* were abundantly expressed in the reproductive organs of *B. xylophilus*. The expression of *Bx-cpi-1* was investigated using qPCR. *Bx-cpi*-*1* was expressed during each of the different developmental stages of *B. xylophilus*. The highest gene expression was at the egg stage. After infection of *Pinus massoniana*, the expression of *Bx-cpi-1* increased. The functions of *Bx-cpi-1* were verified by RNA interference. The feeding rate, reproduction and pathogenicity of *B. xylophilus* all decreased as a result of silencing of the *Bx-cpi-1* gene. These results revealed that *Bx-cpi-1* may be a variant of a type II cystatin gene which is involved in the development and pathogenic process of *B. xylophilus*.

## Abstract (precis)

**Summary:** *Bx-cpi-1*, a variant of a type II cystatin gene, was abundantly expressed in the reproductive organs of *Bursaphelenchus* *xylophilus*, and involved in the development and pathogenicity of *B. xylophilus*.

## INTRODUCTION

The pine wood nematode (PWN), *Bursaphelenchus xylophilus*, is a plant-parasitic nematode and the causal agent of pine wilt disease (PWD). It is native to North America and causes little damage to indigenous tree species (Rautapää, 1986). At the start of the 20th century, it spread to East Asian countries; China, Japan and Korea, and caused significant damage under appropriate environmental conditions (Mamiya, 1988; Yi et al., 1989; Zhou et al., 2017). Subsequently, it was introduced to Nigeria (Khan and Gbadegesin, 1991), Mexico (Dwinell, 1993), Portugal (Burgermeister et al., 1999) and Spain (Abelleira et al., 2011). The disease has become a problem with the potential to cause significant economic losses and damage to forests on an ecological scale (Li et al., 2018). Despite many advances in the study of PWD, the pathogenic mechanism has not been elucidated (Liu et al., 2017).

Cystatins are widespread in all eukaryotes (mammals, nematodes, arthropods, plants, etc.) and participate in various biological processes by regulating normal proteolysis, while also taking part in disease pathology (Zavasnik-Bergant, 2008). They regulate the molting of *Onchocerca volvulus* (Lustigman et al., 1992), protect the intestinal epithelial lining from endogenous proteolysis by cysteine peptidases in *Clonorchis sinensis* (Kang et al., 2014), and contribute to the innate immunity against microorganisms in ectoparasitic arthropods and leeches (Zhou et al., 2006). Many cystatin genes have been predicted from the genomics of plant parasitic nematodes, such as *Bursaphelenchus* (Kikuchi et al., 2011), *Ditylenchus* (Zheng et al., 2016), *Globodera* (Cotton et al., 2014; Eves-van den Akker et al., 2016) and *Meloidogyne* (Opperman et al., 2008; Szitenberg et al., 2017; Somvanshi et al., 2018). However, there have been no detailed reports about these genes.

Compared with the peptidase inhibitors in the secretome of *Meloidogyne incognita* and *Brugia malayi*, the number of peptidase inhibitors secreted by *B. xylophilus* is significantly greater, particularly with respect to cysteine peptidase inhibitors (Shinya et al., 2013). Cysteine peptidases play key roles in plant defense systems and various physiological phenomena (Grudkowska and Zagdańska, 2004; van der Hoorn and Jones, 2004). Cystatins are natural inhibitors of cysteine proteases as a result of tight reversible binding. They are able to defend themselves against host plant cysteine peptidases, as cystatin-type molecules secreted from parasites downregulate the host immune response (Yao and Fu, 2012). Therefore, the study of cysteine protease inhibitors of *B. xylophilus*, a plant-parasitic nematode, will help us to understand the molecular mechanism of PWD.

In the current study, we cloned a full-length cDNA encoding a cystatin of *B. xylophilus* and localized its transcript by *in situ* hybridization, while its expression profiles in *B. xylophilus* at different developmental and pathogenic stages associated with PWD were also analyzed. The functions of *Bx-cpi-1* on the development and pathogenicity of PWN were verified by RNA interference (RNAi). All the data generated provided more clues to deepen our understanding of the *Bx-cpi-1* function in regulating nematode development and pathogenesis.

## RESULTS

### The cloning and sequence analysis of *Bx-cpi-1* in *B. xylophilus*

**Fig. 1.**
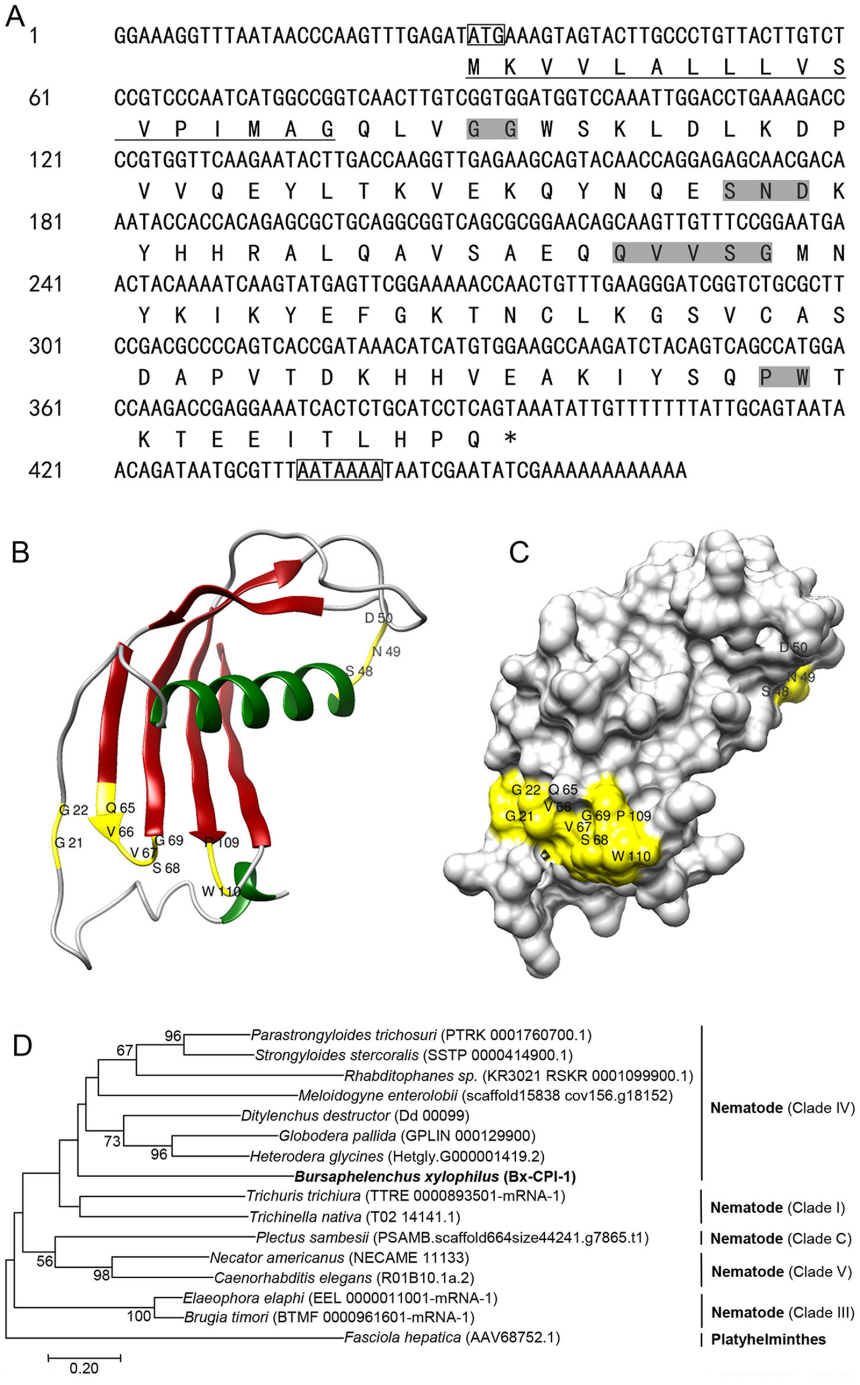
***Bx-cpi-1* sequence analysis.** (A) *Bx-cpi-1* cDNA sequence and its deduced amino acid sequence. The start codon (ATG) and the polyadenylation signal sequences (AATAAA) are indicated in boxes; the underlined area indicates the predicted signal peptide. The highly conserved motifs (GG, SND, QVVSG and PW) are highlighted in grey, while the asterisk (*) indicates the stop codon (TAA). (B) The 3D structure comprises two alpha-helixes and five beta-strands. Motifs critical for binding to peptidases (GG, SND, QVVSG and PW) are highlighted in yellow (the numbering applies to native Bx-CPI-1). (C) Surface structures included in the 3D model. (D) Molecular phylogenetic analysis of Bx-CPI-1 by the Neighbor-Joining method. The phylogram was constructed based on amino acid sequences of 16 CPI proteins using MEGA 7.0. The numbers below the branches indicate the bootstrap values, which are calculated from 1000 replicates. Bootstrapping values are indicated as percentages (when >50%) at the branches. The evolutionary distances were computed using the Poisson correction method and are in the units of the number of amino acid substitutions per site. The accession numbers of the Nematode sequences in brackets are from WormBase ParaSite and the accession number of Platyhelminthes in brackets is from NCBI.

The full-length *B. xylophilus* cystatin sequence was obtained using 3′ RACE and 5′ RACE PCR amplifications. The complete cDNA sequence of *Bx-cpi-1* was 467 bp long, including a 29-bp 5′ untranslated region (*UTR*), a 366-bp open reading frame (ORF), and a 72-bp 3′ *UTR*. It encoded a protein of 121 amino acid residues with a predicted molecular weight of 13.63 kDa and a theoretical isoelectric point (p*I*) value of 7.78. Amino acids 1–17 were not found in the mature Bx-CPI-1 protein and constituted a predicted signal peptide. The amino acid sequence had three conserved regions typical of cystatins, namely a pair of N-terminal glycines (G21G22), a Gln-Val-Val-Ser-Gly motif (Q65VVSG69) in the central part of the molecule, and a Pro-Trp motif (P109W110) in the C-terminal region. The amino acid sequence also had a conserved S48ND50 motif that was shown to constitute a distinct second inhibitory site that was specific for the C13 family of cysteine proteases, which included asparaginyl endopeptidase (AEP), or legumain. However, it was shown to have no disulfide bonds and to be un-glycosylated (Fig. 1A).

Six templates (d1roaa, d1cewi, c4n6oB, c4it7C, c3I0rA and c2ch9A) were selected to model the three-dimensional (3D) structure of Bx-CPI-1 based on heuristics to maximize confidence, percentage identity and amino acid alignment coverage using the Phyre2 web tool. The model of Bx-CPI-1 was built with 89% of the residues having over 90% confidence, whereby 13 residues were modeled by *ab initio*. The theoretical tertiary structure consisted of two alpha helices and five beta-strands (Fig. 1B,C). The predicted 3D structure model was evaluated using the software SAVES. The Laplace image (Ramachandran Plot) showed that there were 81.5% residues in most favored regions, 16.7% residues in allowed regions and 1.9% residues in disallowed regions (Fig. S1A). 64.46% of the residues had averaged 3D-1D score≥0.2 (Fig. S1B). This confirmed that part of the 3D model of Bx-CPI-1 was reasonable. It needed to be further refined, especially the part built by *ab initio*.

The multiple amino acids sequence alignment showed Bx-CPI-1 shared about 35.67% homology with the other 14 nematode cystatins. Almost all the cystatins contained the conserved GG, QxVxG and SNx motif, and cystatins partly have a PW motif (Fig. S2). Phylogenetic analysis was performed to investigate the relationships between Bx-CPI-1 and other cystatins of nematodes. Bx-CPI-1 exhibited a closer relationship with cystatins in nematode clade IV than the cystatins in the other four nematode clades (Fig. 1D).

### Tissue localization of *Bx-cpi-1* in *B. xylophilus*

**Fig. 2.**
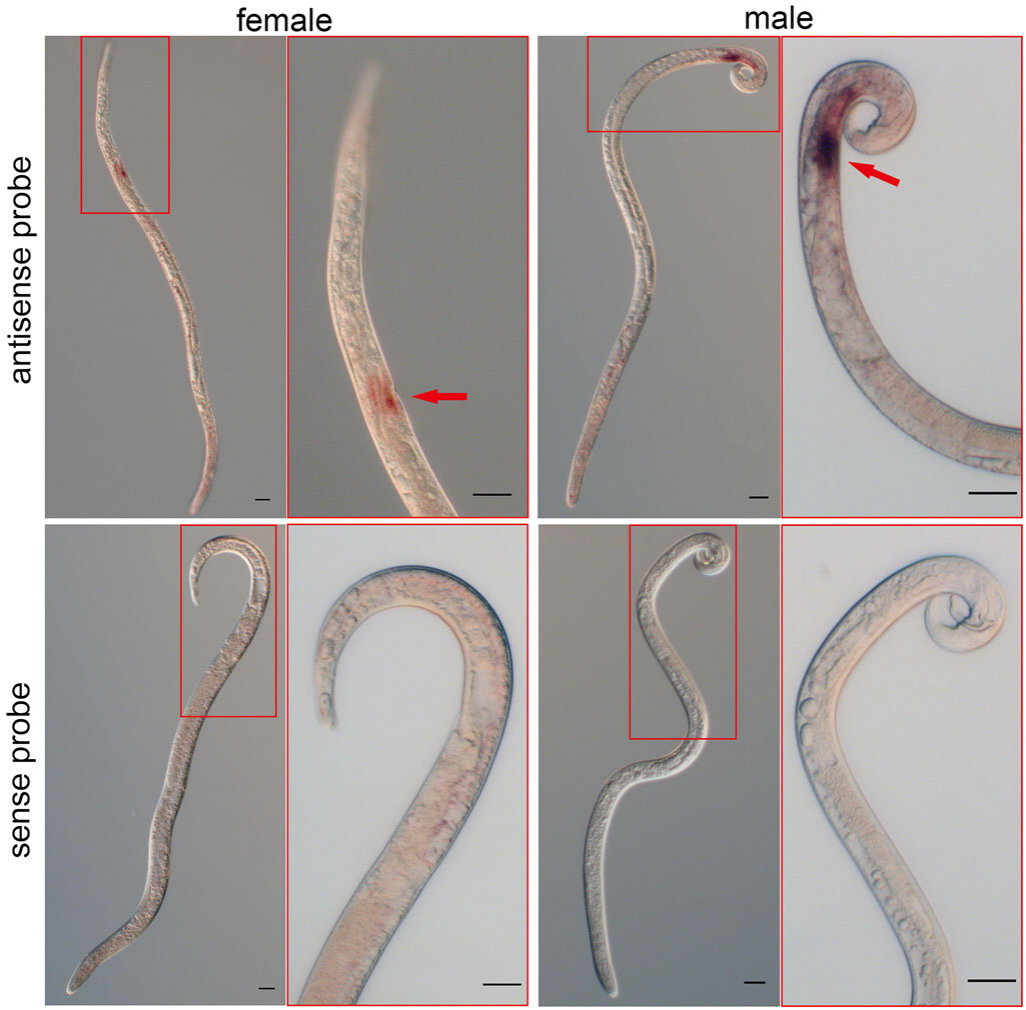
**Localizations of *Bx-cpi-1* mRNA by *in situ* hybridization (ISH).** The hybridization sites are shown in the vulval and uterus regions of female PWNs and the vesicle vas deferens of male PWNs (arrows). The control group shows no signals in females or males. Scale bars: 20 µm.

According to *in situ* hybridization assays, *Bx-cpi-1* was highly expressed in the reproductive organs, with high levels in the vulval region of female and the vas deferens of male of *B. xylophilus* (Fig. 2). No signal was observed in the negative control (Fig. 2).

### Expression of *Bx-cpi-1* at different developmental stages of *B. xylophilus*

**Fig. 3.**
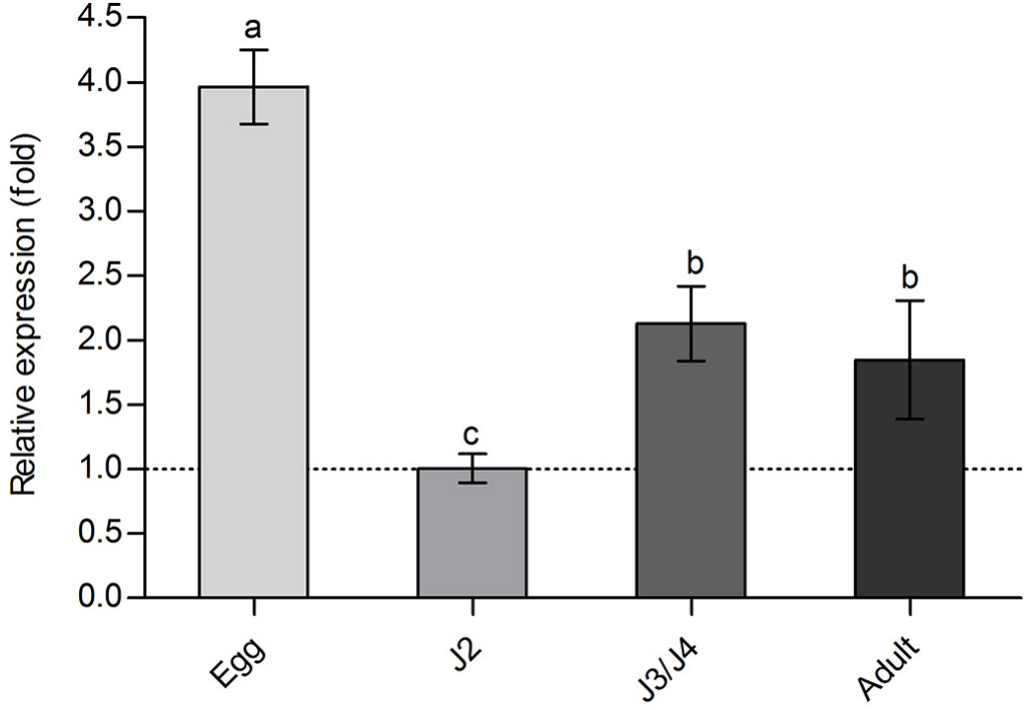
**Relative expression levels of *Bx-cpi-1* at different developmental stages of *B. xylophilus*.** The data are presented as mean±s.d. of three biological replicates and three technical replicates (*n*=9). The reference gene is *actin*. The bars indicate s.d., and any two samples with a common letter are not significantly different (*P*>0.05) according to LSD tests.

The stage-specific expression of *Bx-cpi-1* transcripts was analyzed by qPCR. *Bx-cpi-1* was differently expressed in the nematodes of different ages. Using the expression level in J2 as reference, *Bx-cpi-1* was significantly (*P*<0.05) upregulated at the egg, mixed J3/J4 and adult stages, with maximum expression at the egg stage (Fig. 3). The speculation was that *Bx-cpi-1* may play an essential role in the egg stage of *B. xylophilus*.

### Expression characteristics of *Bx-cpi-1* after *Pinus massoniana* inoculated by *B. xylophilus*

**Fig. 4.**
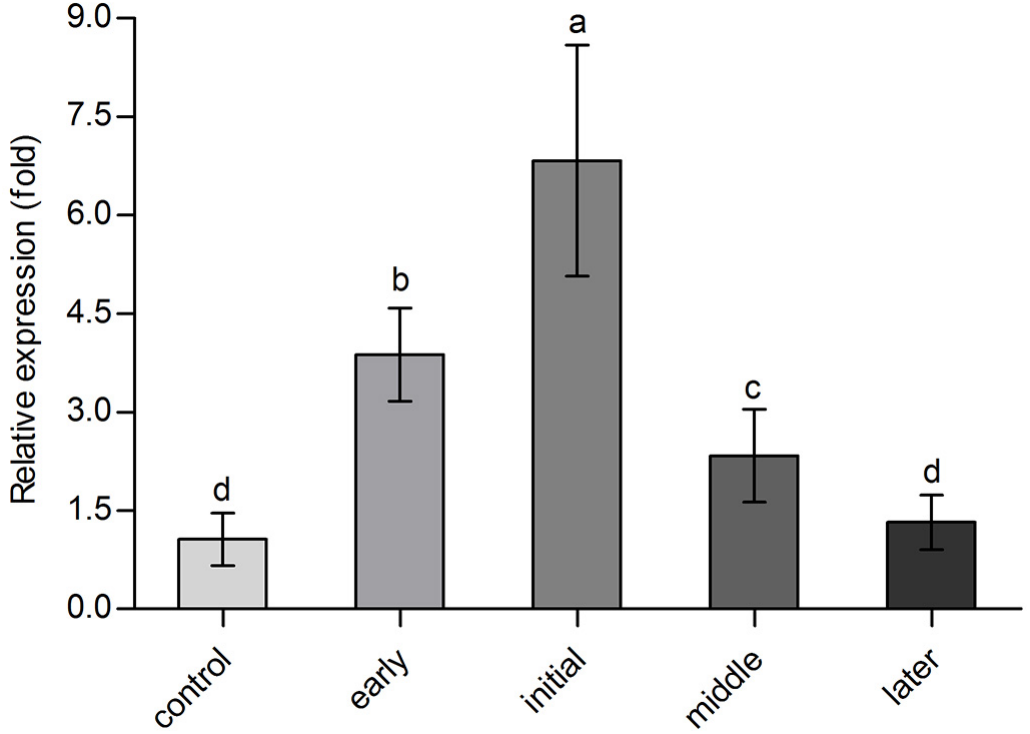
**Relative expression levels of *Bx-cpi-1* after inoculation.** The data are presented as mean±s.d. of three biological replicates and three technical replicates (*n*=9). The reference gene is *actin*. The control group is the *B. xylophilus* cultivated on *B. cinerea*. The bars indicate s.d., and any two samples with a common letter are not significantly different (*P*>0.05) according to LSD tests.

The expression of *Bx-cpi-1* in *B. xylophilus* in two growth conditions: growth on *Botrytis* *cinerea* and after inoculating *P. massoniana* with PWNs, were detected to see if the gene was involved in parasitism. Compared with the expression level of *Bx-cpi-1* in *B. xylophilus* cultured on *B. cinerea* (control, CK), expression increased after *B. xylophilus* was inoculated on to *P. massoniana*. In addition, the expression of *Bx-cpi-1* in *B. xylophilus* after inoculation at different PWD development were detected. The relative expression of *Bx-cpi-1* was higher when no visible symptoms were registered. At the initial stage of PWD, *Bx-cpi-1* expression peaked when the pines began to show external symptoms (Fig. S3). As the degree of damage increased gradually, more and more needles turned brown, and the *Bx-cpi-1* expression decreased in the middle and later stages of PWD (Fig. 4).

### Feeding and reproduction of *B. xylophilus* after RNAi

**Fig. 5.**
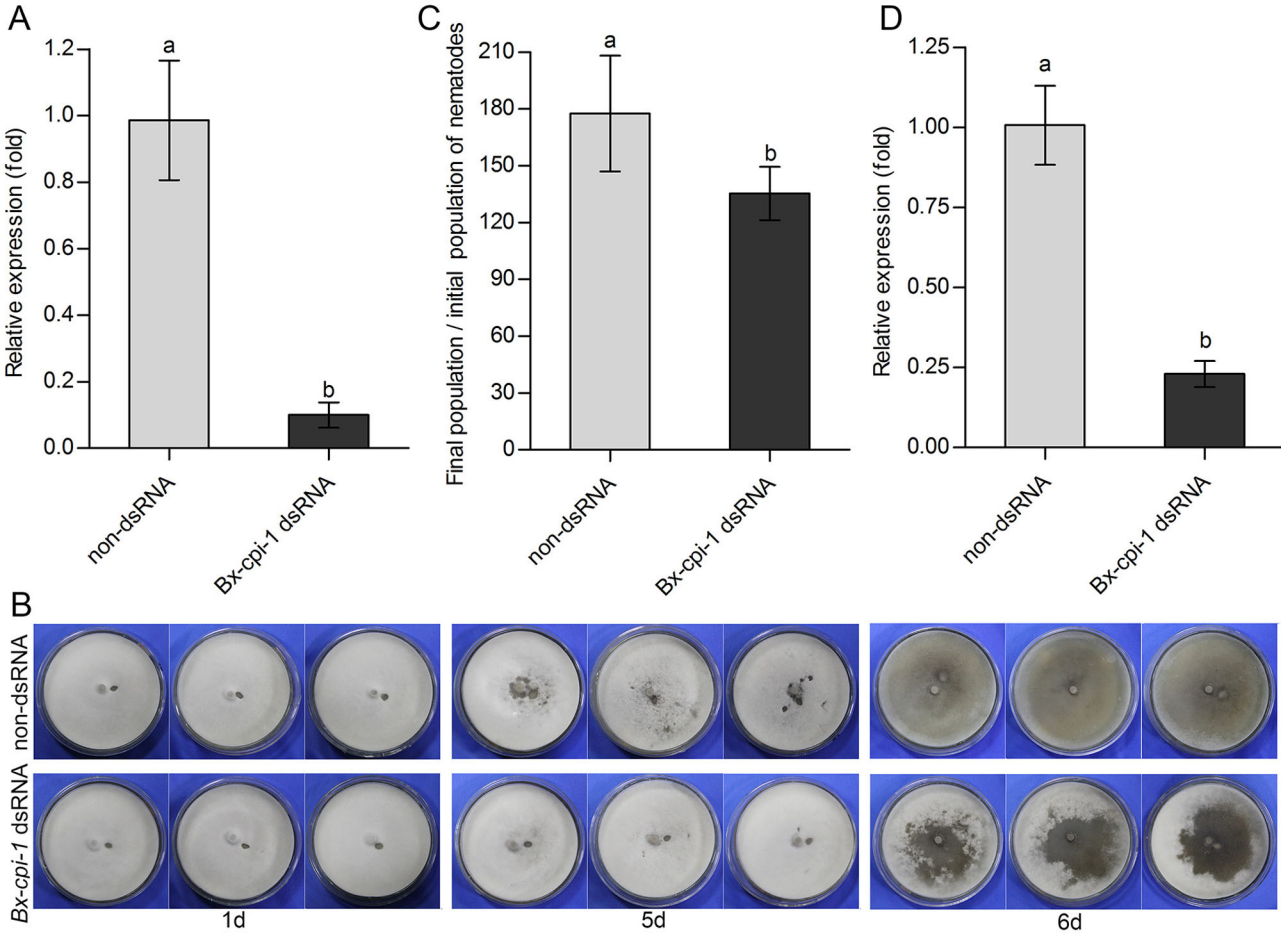
**Effects of RNAi on *Bx-cpi-*1 expression, feeding and reproduction by *B. xylophilus*.** (A,B) Relative expression levels of *Bx-cpi-1* after treatment with *Bx-cpi-1* dsRNA solution (A); RNAi-treated *B. xylophilus* after cultivation on *B. cinerea* (B). (C) Total number of *B. xylophilus* recovered from *B. cinerea* plates 6 days after treatment with non-dsRNA solution and *Bx-cpi-1* dsRNA solution. (D) Relative expression levels of *Bx-cpi-1* after cultivation on *B. cinerea* for 6 days. The bars indicate s.d. of three biological replicates and three technical replicates (*n*=9), and different letters indicate significant differences (*P*<0.05) among treatments within a bar chart, using a Student's *t*-test.

After soaking in dsRNA solution, the nematodes in treatment and control group were all alive, and there was no visible difference in morphology (Fig. S4). Compared with the control group, the transcript level of *Bx-cpi-1* in the treatment group decreased 90.03% (Fig. 5A). This finding showed that RNAi could be performed by directly soaking the nematodes in the dsRNA solution. The effect of RNAi on the feeding and reproduction of *B. xylophilus* was tested on PDA plates inoculated with *B. cinerea*. *Bursaphelenchus* *xylophilus* treated with non-dsRNA solution fed faster than those treated with *Bx-cpi-1* dsRNA solution (Fig. 5B). It took about 5 days to complete a generation of nematodes, so the production of nematodes was analyzed at 6 days. The reproduction rates of nematodes in the control group were about 177.56-fold, while the reproduction rates of nematodes in the *Bx-cpi-1* dsRNA treatment group were only 135.33-fold (Fig. 5C). At this time, the transcript level of *Bx-cpi-1* in the *Bx-cpi-1* dsRNA-treated nematodes was still as low as 22.98% (Fig. 5D). The results showed that nematode treatment with *Bx-cpi-1* dsRNA significantly reduced the reproduction of *B. xylophilus* (*P*<0.05).

### Pathogenicity of *B. xylophilus* after RNAi

The 1-year-old and 2-year-old *P. massoniana* seedlings were inoculated with nematodes to test the virulence of nematodes after RNAi (Fig. S5). The 1-year-old *P. massoniana* seedlings inoculated with nematodes treated with non-dsRNA-1 solution exhibited leaf yellowing after 22 days, while the seedlings inoculated with nematodes treated with *Bx-cpi-1* dsRNA-1 showed symptoms after 26 days. The 2-year-old pine seedlings inoculated with nematodes treated with non-dsRNA-2 solution showed symptoms of PWD after 16 days, while the seedlings inoculated with nematodes treated with *Bx-cpi-1* dsRNA-2 showed symptoms after 21 days. Twenty-eight days after inoculation, the infection rates in non-dsRNA-1 and non-dsRNA-2 treatments were 66.6% and 100%, and those in *Bx-cpi-1* dsRNA-1 and *Bx-cpi-1* dsRNA-2 treatment were 66.6% and 75%. The disease severity index (DSI) in non-dsRNA-1 and non-dsRNA-2 treatments were 41.6 and 100, and those in *Bx-cpi-1* dsRNA-1 and *Bx-cpi-1* dsRNA-2 treatment were 25 and 31.25 (Table 1). These results showed that, with the pine seedlings inoculated with nematodes, the onset of PWD was delayed after treatment with *Bx-cpi* dsRNA, which reduced expression of the *Bx-cpi-1* gene.

**Table 1.**
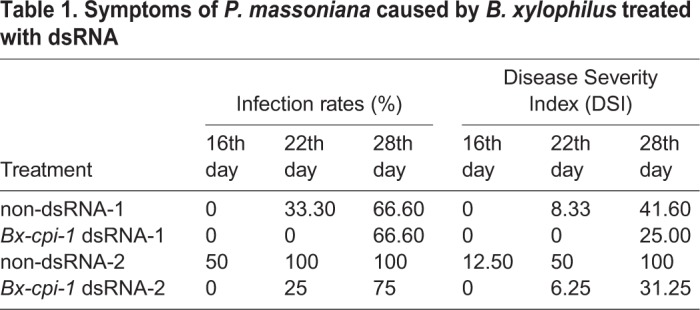
**Symptoms of *P. massoniana* caused by *B. xylophilus* treated with dsRNA**

## DISCUSSION

Cystatins are the reversible inhibitors of cysteine proteinases. Their interaction is through a well-established mechanism, in that the conserved motifs of cystatins form a wedge-shaped edge to completely cover the cysteine peptidase active site cleft (Bode et al., 1988; Stubbs et al., 1990). Cystatins are widely distributed in all eukaryotes and involved in various biological and pathological processes (Honey and Rudensky, 2003; Kos and Lah, 1998). Cystatins evolved during the co-evolution of the parasites and their hosts. Therefore, cystatins of parasitic nematodes and the free-living nematode, *Caenorhabditis elegans*, have different immunomodulatory properties (Hartmann and Lucius, 2003). To date, there are far more studies on characterizing cystatins of filarial nematodes and gastrointestinal nematodes than those of plant-parasitic nematodes (Yao and Fu, 2012). Some cystatin genes of plant-parasitic nematodes, including *Bursaphelenchus*, *Ditylenchus*, *Globodera* and *Meloidogyne*, could be predicted from their genomics (Kikuchi et al., 2011; Zheng et al., 2016; Eves-van den Akker et al., 2016; Somvanshi et al., 2018). However, the functions of these cystatins need to be further studied.

In this study, the full-length cDNA of *Bx-cpi-1* was cloned. Bx-CPI-1, the deduced protein of *Bx-cpi-1*, displays the typical length of a type II cystatin (121 amino acids). It contained three conserved cystatin motifs and a SND motif, which were similar to those of several type II cystatins found in many taxa, which have the ability to inhibit not only peptidases of family C1, but also legumain (family C13) (Alvarez-Fernandez et al., 1999; Martinez et al., 2007). Moreover, it had a predicted signal peptide and was non-glycosylated, a situation, which is consistent with type II cystatins (Bode et al., 1988; Zhang and Lin, 2005). However, it lacked disulfide bonds, whereas typical type II cystatins contain disulfide bonds (Zhang and Lin, 2005). *In silico* 3D modeling revealed substantial structural similarities between Bx-CPI-1 and the cystatins in other species. The phylogenetic relationships among cystatins showed that Bx-CPI-1 clustered together with the cystatins in nematode clade IV. It agreed with the classification of *B. xylophilus* in nematode (Blaxter et al., 1998). These results suggest that Bx-CPI-1 might be a variant of type II cystatin in *B. xylophilus*, which does not contain disulfide bonds.

Unlike the ubiquitous expression of cystatin, the cystatin-related epididymal spermatogenic protein (CRES) may perform tissue-specific functions, resulting from a lack of critical consensus sites of cystatin (Hsia and Cornwall, 2003). In the current study, ISH was used to investigate the localization of *Bx-cpi-1* in *B. xylophilus*. The *Bx-cpi-1* gene was highly expressed in the reproductive organs of both sexes, namely the vulval region of female and the vas deferens of male *B. xylophilus*, according to the structures of reproductive systems of plant nematodes described by Feng (2000). It indicated that *Bx-cpi-1* gene expression exhibited tissue specificity. The *Cres* gene, which is preferentially expressed in post-meiotic germ cells, the proximal caput epididymidis and the anterior pituitary gonadotrophs, plays a role in regulation of prohormone and proprotein processing. Cystatin C (CST3), a cysteine protease inhibitor highly expressed in female and male reproductive tracts of human beings, is important in sperm physiology (Lee et al., 2018). Therefore, it was speculated that *Bx-cpi-1* is involved in the reproduction of *B. xylophilus*.

The prototypical type II cystatin was discovered in chicken egg white (Fossum and Whitaker, 1968). In the present study, the mRNA transcripts of *Bx-cpi-1* could be detected at different developmental stages, with the highest expression level at the egg stage. Rangel et al. (2017) detected the novel cystatins transcripts in different *Ixodes persulcatus* instars, showing their ubiquitous expression during *I. persulcatus* development. Wang et al. (2015) found a cystatin (RHcyst-1) from the tick *Rhipicephalus haemaphysaloides* was more richly transcribed in the embryo (egg) stage, and hypothesized that it plays a role in early embryonic development. It has been proved that the protease inhibitor in eggs protects the eggs from microorganism infection, and plays a regulatory role in the process of embryogenesis and early embryo growth (Guo et al., 2011). It was inferred that *Bx-cpi-1* may play an important role in the egg stage of *B. xylophilus*.

Compared with the expression level of *Bx-cpi-1* in *B. xylophilus* cultured on *B. cinerea*, expression of this gene increased after inoculation on *P. massoniana* seedlings. After pathogen invasion, plants exhibit an oxidative burst as part of the anti-microbial defense mechanism (Torres et al., 2006). Cystatins have exhibited a protective effect against oxidative stresses (Nishiyama et al., 2005). The relative expression levels of *Bx-cpi-1* was highest when pines expressed external symptoms but declined during the development of PWD. This may be related to functions of cystatins in the modulation of normal proteolytic processes and the balance of the host–parasite interaction (Abrahamson et al., 2003; Van Eijk et al., 2003; Zhou et al., 2006).

RNA interference (RNAi) is a powerful technique for gene silencing resulting from treatment with dsRNA. It has been achieved in *B. xylophilus* and other organisms in order to examine the functions of genes (Cardoso et al., 2015; Urwin et al., 2002; Xu et al., 2015). Some studies showed that the target gene's expression was affected by the dsRNA solution of the target gene, rather than the non-dsRNA solution or dsRNA solution of exogenous gene (*gfp*). There was no significant difference between the nematodes soaked in non-dsRNA solution and the dsRNA solution of exogenous gene (Xue et al., 2019; Hu et al., 2018). Therefore, if the target gene's expression of the nematode treated with dsRNA solution of the target gene was significantly lower than that treated with non-dsRNA solution, the interference of the target gene could indicate success. In this study, the expression of *Bx-cpi-1* significantly decreased after *B. xylophilus* individuals were soaked in *Bx-cpi-1* dsRNA solution, implying that the *Bx-cpi-1* gene had been silenced effectively. Silencing of the *Bx-cpi-1* gene with RNAi did not visibly affect the survival and morphology of *B. xylophilus*, but reduced feeding ability and reproduction of the nematodes. Furthermore, it caused a delay in the expression by *P. massoniana* symptoms. These observed phenotypes were due to the direct effect of the interference of *Bx-cpi-1* gene. It has been reported that the virulence of *B. xylophilus* is weakly correlated with its propagation rate on *B. cinerea* (Zhu et al., 2015). Therefore, *Bx-cpi-1* might play regulatory roles in the development and pathogenicity of *B. xylophilus*. This study focused on the molecular characterization and functional analysis of *Bx-cpi-1*, showing that *Bx-cpi-1* might play a role in the development and pathogenicity of *B. xylophilus*. The results of this study might provide useful information for a better understanding of the molecular mechanism of reproduction and pathogenicity in *B. xylophilus*.

## MATERIALS AND METHODS

### Nematode culture and collection

The *B. xylophilus* isolate (AMA3) was collected from woodchips of infested *P.* *massoniana* in Maanshan, Anhui, China. It was provided by the Jiangsu Key Laboratory for Prevention and Management of Invasive Species, Nanjing Forestry University.

Mixed-stage nematodes, including eggs, 2nd-stage juveniles (J2), 3rd-stage juveniles (J3), 4th-stage juveniles (J4) and adults were cultured on the fungus *B.* *cinerea* grown on potato dextrose agar (PDA) at 25°C for 5 days. The nematode eggs were collected from the culture according to the method described by Iwahori and Futai (1985) and incubated in sterilized distilled water at 25°C. After 2 days, a large number of hatched J2s were collected and transferred to a PDA plate culture of *B. cinerea*. The mixed J3s/J4s were collected at 30–48 h, and the adults were collected at 78 h after re-initiation of feeding (Shinya et al., 2009). PWNs at each developmental stage were identified under a 10−40× optical microscope (Leica DM500; Leica Microsystems, Heerbrugg, Switzerland) and then used in subsequent experiments. The nematodes of each stage were collected until the numbers in each sample reached 3000. This process was performed three times.

Approximately 10,000 mixed-stage nematodes were used to inoculate 2-year-old potted *P. massoniana* seedlings. Twenty-four inoculated seedlings were incubated in the greenhouse at 25°C. The PWD development was divided into four distinct stages as follows: (1) the early stage, all needles were green; (2) the initial stage, needles began to turn brown; (3) the middle stage, about half of the needles turn brown; and (4) the later stage, needles were completely brown. Photographs were taken at regular intervals to record the infection state of the seedlings (Fig. S3). Every time, the PWN population that was extracted from woody pieces of two inoculated seedlings at different PWD development stages using the Baermann funnel technique was as a biological repeat. Each stage of PWD included three biological replicates.

Prior to RNA extraction, nematodes were washed three times with sterilized distilled water and suspended in a 1.5-ml microtube (Eppendorf) in a minimal amount of water, before being stored at −80°C after freezing in liquid nitrogen.

### RNA isolation and cDNA synthesis

Total RNAs were extracted from each nematode sample using the TRIzol reagent (Invitrogen, Waltham, MA, USA). The RNA concentration was measured by ultraviolet absorbance NanoDrop 2000C at A260/280 (Thermo Fisher Scientific, Wilmington, Delaware, USA) and the quality was assessed by electrophoresis on a 1% agarose gel. The first-strand cDNA was synthesized from 1 µg total RNA using the HiScript^®^ II Q RT SuperMix for qPCR (+gDNA wiper) (Vazyme, Nanjing, China), which could completely remove the residual genomic DNA in the RNA template.

### Cloning of *Bx-cpi-1* gene

The full-length *Bx-cpi-1* cDNA was obtained by rapid-amplification of cDNA ends (RACE) using the 3′-Full RACE Core Set with the PrimeScript™ RTase kit and 5′-Full RACE Kit with TAP (TaKaRa Biotechnology, Dalian, China) following the manufacturer's instructions. The 3′ RACE outer primer (3′-*Bx-cpi-1-*R1), 3′ RACE inner primer (3′-*Bx-cpi-1-*R2), 5′ RACE outer primer (5′-*Bx-cpi-1-*F1) and 5′ RACE inner primer (5′-*Bx-cpi-1-*F2) were provided in the kits. Gene-specific primers (3′-*Bx-cpi-1-*F1, 3′-*Bx-cpi-1-*F2, 5′-*Bx-cpi-1-*R1 and 5′-*Bx-cpi-1-*R2) (Table 2) were designed for 3′ and 5′ RACE amplification based on partially known sequences of *Bx-cpi-1*, which were obtained from the RNA sequencing results (He et al., 2016). Four primer pairs 3′-*Bx-cpi-1-*F1 / 3′-*Bx-cpi-1-*R1, 3′-*Bx-cpi-1-*F2 / 3′-*Bx-cpi-1-*R2, 5′-*Bx-cpi-1-*F1 / 5′-*Bx-cpi-1-*R1, and 5′-*Bx-cpi-1-*F2 / 5′-*Bx-cpi-1-*R2 were used for 3′-Full RACE first round of PCR, 3′-Full RACE second round of PCR, 5′-Full RACE first round of PCR and 5′-Full RACE second round of PCR, respectively. The amplification profiles were all as follows: a cycle at 94°C for 3 min, 30 cycles at 94°C for 30 s, 55°C for 30 s, 72°C for 45 s and 72°C for 10 min. The resulting nested PCR product was purified and ligated into the pEASY-T1 vector (TransGen Biotech, Beijing, China) and transformed into *Escherichia coli* Trans1-T1 competent cells (TransGen Biotech). The *E. coli* was then incubated overnight at 37°C on LB plates containing ampicillin. After PCR detection with primers M13F (−47)/M13R (−48) (Table 2), the fresh bacterial suspension was sequenced at Nanjing Genscript Sequencing Company (Nanjing, China). The full-length cDNA sequence of *Bx-cpi-1* was submitted to GenBank as the accession number MK348536.

**Table 2.**
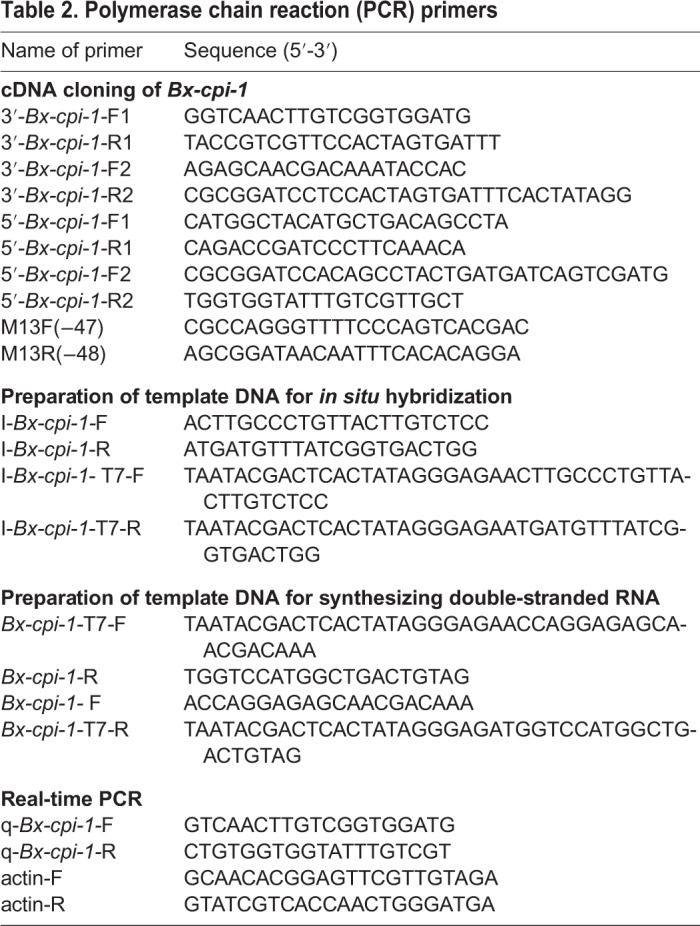
**Polymerase chain reaction (PCR) primers**

### Bioinformatic analysis

The ORF of *Bx-cpi-1* was identified by ORF Finder (https://www.ncbi.nlm.nih.gov/orffinder/). Deduction of the amino acid sequence, calculation of theoretical molecular mass and the theoretical value was performed with the ExPASy ProtParam online tool. SignalP 4.1 (http://www.cbs.dtu.dk/services/SignalP/) was used for signal peptide prediction. Prediction of the secondary and tertiary structures was carried out using the Phyre2 online tool (http://www.sbg.bio.ic.ac.uk/phyre2/html/page.cgi?id=index) set to intensive mode (Kelley et al., 2015). Subsequent molecular graphics and analyses were performed using UCSF Chimera v. 1.10.2 (Pettersen et al., 2004). The predicted tertiary structure model was assessed using online software SAVES v5.0 (http://servicesn.mbi.ucla.edu/SAVES/) (Gong et al., 2015).

Homologous sequences of the Bx-CPI-1 protein were obtained from WormBase ParaSite using blastp (https://parasite.wormbase.org/Multi/Tools/Blast?db=core) and the outgroup protein sequences were obtained from NCBI (https://www.ncbi.nlm.nih.gov). Multiple sequence alignment of deduced protein sequences was carried out with DNAMAN 6.0. The ClustalW method implemented in MEGA 7.0 was used to align amino acid sequences prior to phylogenetic analysis (Kumar et al., 2016). The evolutionary history was inferred by using the Neighbor-Joining method (Saitou and Nei, 1987). The evolutionary distances were computed using the Poisson correction method (Zuckerkandl and Pauling, 1965). The analysis involved ten cysteine protease inhibitor (CPI) amino acid sequences. All positions containing gaps and missing data were eliminated. Evolutionary analyses were conducted in MEGA7.0 (Kumar et al., 2016).

### ISH

ISH enables the study of gene expression patterns *in situ* within nematodes (Deng et al., 2016; Hashmi et al., 2002; Shingles et al., 2007). The ISH probe templates were generated by PCR based on the full-length cDNA sequences of *Bx-cpi-1* with the specific primer pairs (Table 2). The digoxigenins (DIG)-labeled sense RNA probes and antisense RNA probes were synthesized using the DIG Northern Starter Kit (Roche Diagnostics, Mannheim, Germany) (Regina et al., 2012). The control group used the DIG-labeled sense RNA probe. The nematodes were treated and hybridizations were performed as described by De Boer et al. (1998) and Cheng et al. (2013) using DIG High Prime DNA Labeling and Detection Starter Kit I (Roche Diagnostics, Mannheim, Germany). Finally, the nematodes were examined and photographed using a Zeiss Axio Image M2 microscope (Zeiss MicroImaging GmbH, Oberkochen, Germany).

### Synthesis of *Bx-cpi-1* dsRNA and interference RNA

Based on the *Bx-cpi-1* sequence, two primer pairs *Bx-cpi-1-*T7*-*F/*Bx-cpi-1-*R and *Bx-cpi-1-*F/*Bx-cpi-1*-T7-R (Table 2) were designed to amplify the sense and anti-sense single stranded RNA (ssRNA) products with the MEGAscript RNAi Kit (Ambion Inc., Austin, TX, USA). The two ssRNAs obtained were mixed at 75°C for 5 min and cooled to room temperature. After purification, the quality of the double-stranded RNA (dsRNA) was checked on a 1% agarose gel and quantified by spectrophotometry. RNA interference (RNAi) was carried out on mixed-stage nematodes using the procedures described by Urwin et al. (2002). Approximately 3000 freshly cultured nematodes were soaked in dsRNA solution (1 µg/µl) after being washed with distilled water three times at 3500×***g*** for 3 min. Nematode suspensions were shaken slowly (180 rpm) on a shaking table incubated at 20°C for 48 h. Nematodes soaked in the corresponding non-dsRNA solution were used as controls. Each treatment had three replicates. After soaking, the samples of each treatment were washed thoroughly several times using ddH_2_O to remove external dsRNA, and then used for further experiments.

### Gene expression analysis by qPCR

RT-qPCR was used to assess the gene expression patterns of *Bx-cpi-1*. About 80 ng cDNA of each sample was used as the template to perform qPCR with a SYBR Green Master Mix (Vazyme, Nanjing, China) on the ABI Prism 7500 Real-Time PCR System (Applied Biosystems, Foster City, CA, USA). The primers q-*Bx-cpi-1-*F and q-*Bx-cpi-1-*R were designed for amplification using Primer Premier 5 (PREMIER Biosoft International, Palo Alto, CA, USA). The housekeeping *actin* gene of *B. xylophilus* was utilized as the internal control gene, using primers actin-F/R obtained from NCBI with probe accession number Pr031904943, for calculation of relative expression levels of the cystatin gene (Deng et al., 2016). Thermal cycling conditions were as follows: initial denaturation at 95°C for 5 min, 40 cycles of denaturation at 95°C for 10 s, annealing and extension at 60°C for 34 s; followed by the melting curve. A single peak at the melting temperature of the PCR product confirmed primer specificity. Experiments were repeated three times. Relative gene expression was analyzed by the ΔΔC_q_ method with 7500 software v2.3 (Life Technology Corporation, Carlsbad, USA). The standard curve and melting curves of *actin* and *Bx-cpi-1* were shown in Fig. S6.

### Analysis of reproduction and pathogenicity of *B. xylophilus* after RNAi

About 100 nematodes treated with non-dsRNA solution and *Bx-cpi-1* dsRNA solution were transferred to a PDA plate culture of *B. cinerea* and incubated at 25°C for 6 days. The feeding of *B. xylophilus* was observed and photographed periodically. The nematodes were washed from the plates using the Baermann funnel technique and counted with an optical microscope (Leica DM500, Leica Microsystems, Heerbrugg, Switzerland). Each treatment had three replicates. The remaining nematodes were used for inoculation assays. Each 1-year-old/2-year-old *P. massoniana* seedling was inoculated with approximately 500/1500 mixed-stage nematodes. Inoculation using ddH_2_O without nematodes was used as the control. The inoculated seedlings were grown in the greenhouse at 25°C. PWD symptoms were evaluated and categorized as 0–4 (Yu et al., 2012). The categories were as follows: 0=all needles were green; 1=0–25% of needles were discolored and turning yellow; 2=26–50% of needles had turned yellow; 3=51–75% of needles had turned yellow; and 4=76–100% of needles had turned yellow. The infection rates and the disease severity index (DSI) were calculated with the equations as follows:
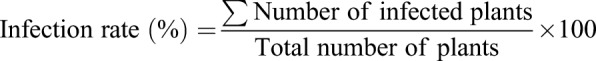

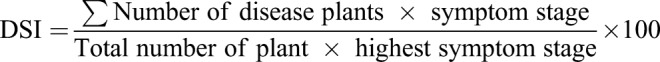


### Statistical analysis

The data were presented as means±s.d. of three independent experiments. All parameters were calculated using Microsoft Excel and GraphPad Prism 5 (GraphPad Software, San Diego, CA, USA). The statistical significance was determined using SPSS Statistics 24.0 software (IBM China Company Ltd., Beijing, China). A Student's *t*-test was used to compare two samples (Fig. 5); ANOVA plus a Least Significant Difference (LSD) test was used to compare more than two samples (Figs 3 and 4). The level of significance was *P*<0.05.

## Supplementary Material

Supplementary information
