# Supplementary material for: Characteristics and function of a novel cystatin gene in the pine wood nematode Bursaphelenchus xylophilus
Source: Biol Open. 2019 Sep 11;8(9):bio042655. doi: 10.1242/bio.042655 (PMC6777362; doi:10.1242/bio.042655)
Supplement: Supplementary information [file biolopen-8-042655-s1.pdf]

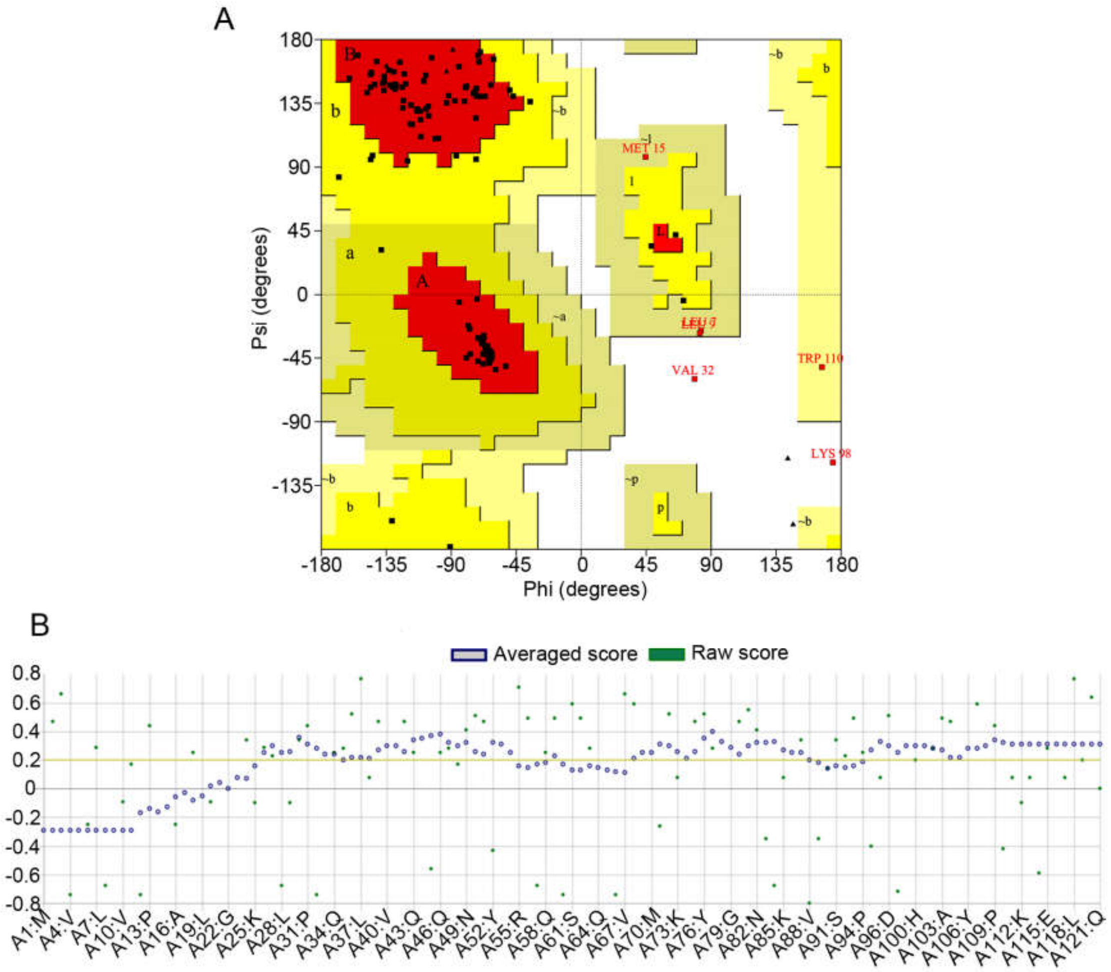

Fig. s1. The predicted 3D structure assessment of Bx-CPI-1. Laplace image (Ramachandran Plot) of 3D structure of Bx-CPI-1 (**A**). Residues in most favoured regions [A,B,L] are 88 (81.5%); Residues in additional allowed regions [a,b,l,p] are 14 (13.0%); Residues in generously allowed regions [~a,~b,~l,~p] are 4 (3.7%); Residues in disallowed regions are 2 (1.9%). Number of non-glycine and non-proline residues is 108; Number of end-residues (excl. Gly and Pro) is 2; Number of glycine residues (shown as triangles) is 6; Number of proline residues is 5. Total number of residues is 121. Verify 3D model of Bx-CPI-1 (**B**). The 66.99% of the residues have averaged 3D-1D score ≥ 0.2.

Biology Open • Supplementary information

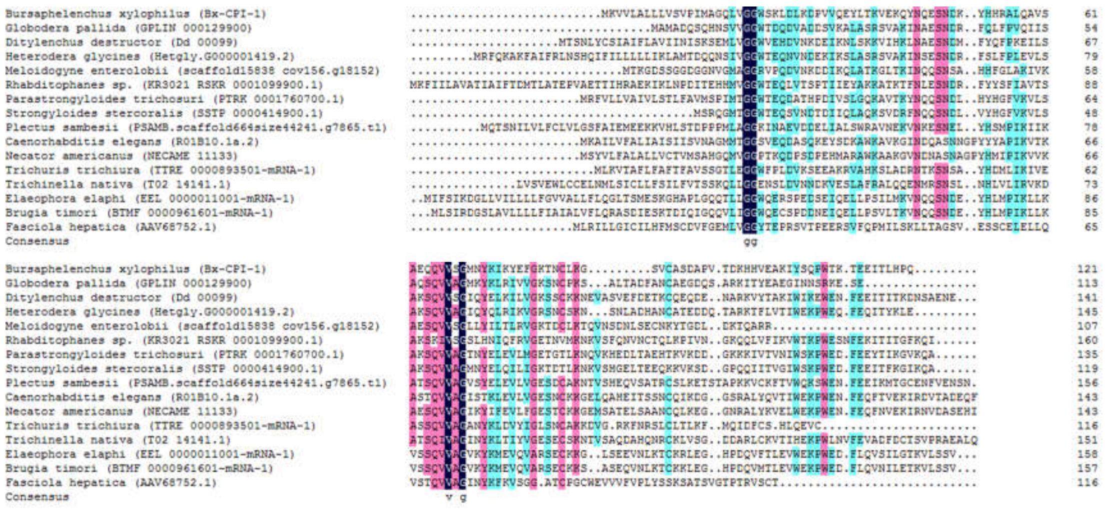

Fig. S2. Multiple alignments of predicted Bx-CPI-1 protein sequence of *B. xylophilus* with other 15 cystatins. Letters in black boxes, red boxes, and blue boxes indicate invariant amino acid residues, highly conserved amino acid residues, and moderately conserved amino acid residues, respectively, among the CPI proteins.

Biology Open • Supplementary information

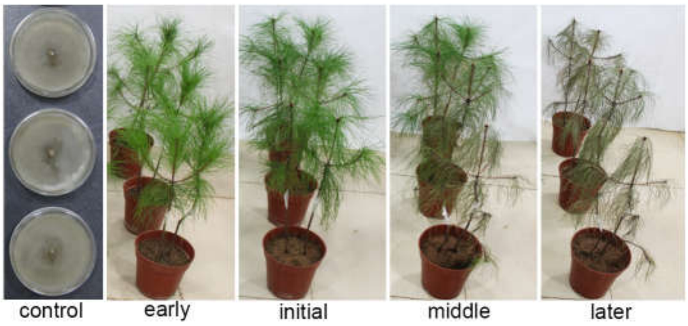

Fig. S3. Symptoms in *P. massoniana* after inoculation with *B. xylophilus*. Each seedling was inoculated with about 10,000 nematodes. The early stage of PWD (early); the initial stage of PWD (initial); the middle stage of PWD (middle); the later stage of PWD (later); the nematodes cultured on *B. cinerea* (control).

Biology Open • Supplementary information

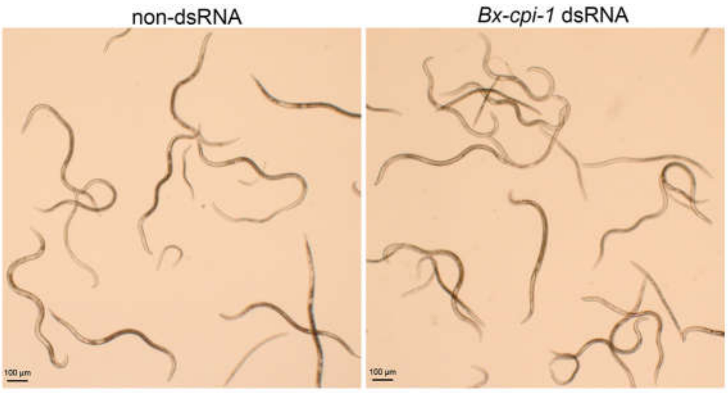

Fig. S4. Morphology of *B. xylophlilus* after soaking in dsRNA (non-dsRNA and *Bx-cpi-1* dsRNA) for 48 h. Scale bars = 100 μm.

Biology Open • Supplementary information

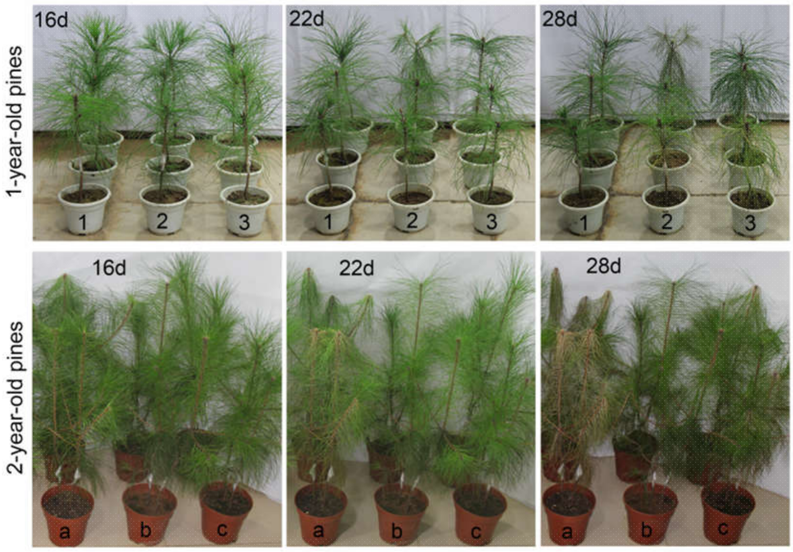

Fig. S5. Symptoms in *P. massoniana* after inoculation with *B. xylophilus*. The 1-year-old pines inoculated with 500 nematodes, and 2-year-old pines inoculated with 1,500 nematodes. Photographs shows the symptoms at 16d, 22d and 28d. Within each photograph, pot 1 and b indicates pines inoculated with ddH_2O, pot 2 and a indicates pines inoculated with *B. xylophilus* soaked in non-dsRNA, and pot 3 and c indicates pines inoculated with *B. xylophilus* soaked in *Bx-cpi-1* dsRNA.

Biology Open • Supplementary information

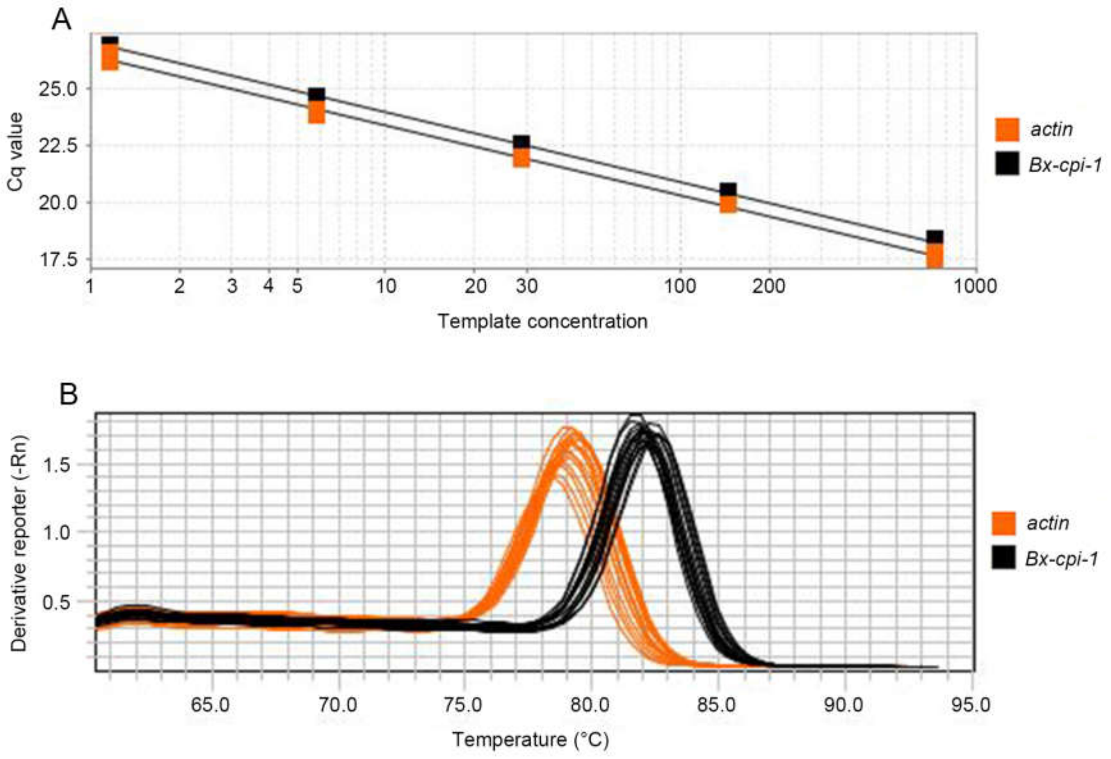

Fig. S6. The standard curve and melt curve of *actin* and *Bx-cpi-1*. The standard curve of *actin* and *Bx-cpi-1* (**A**). Cq value is proportional to template cDNA concentration. The slope of *actin* and *Bx-cpi-1* was -3.058 and -3.071, the correlation coefficient (R^2) of *actin* and *Bx-cpi-1* was 0.998 and 0.999, the amplification efficiency of *actin* and *Bx-cpi-1* was 112.356% and 111.659%. The melt curve of *actin* and *Bx-cpi-1* (**B**).

Biology Open • Supplementary information
